# Distance Measurement of Contra-Rotating Rotor Blades with Ultrasonic Transducers

**DOI:** 10.3390/mi15060676

**Published:** 2024-05-22

**Authors:** Shan Zhang, Yaohuan Lu, Zhen Qiu, Wenchuan Hu, Zewen Dong, Zurong Qiu, Yongqiang Qiu

**Affiliations:** 1 State Key Laboratory of Precision Measuring Technology and Instruments, Tianjin University, Tianjin 300072, China; 2 School of Engineering, University of Bolton, Bolton BL3 5AB, UK; 3 College of Mechanical Engineering, Tianjin University of Technology and Education, Tianjin 300355, China; 4 School of Engineering, Liverpool John Moores University, Liverpool L3 3AF, UK

**Keywords:** rotor blade distance, ultrasonic ranging, bandpass filtering, TOF estimation

## Abstract

Coaxial rotor helicopters have great potential in civilian and commercial uses, with many advantages, but challenges remain in the accurate measurement of rotor blades’ distance to prevent blade collision. In this paper, a blade tip distance measurement method based on ultrasonic measurement window and phase triggering is proposed, and the triggering time of the transmitter is studied. Due to the complexity of the measured signal, bandpass filtering and a time-of-flight (TOF) estimation based on the power density of the received signal are utilised. The method is tested on an experimental test platform with a pair of 200 kHz ultrasonic transducers. The experimental results show that the maximum ranging error is less than 1.0% for the blade tip distance in a range of 100–1000 mm. Compared with the amplitude threshold method, the proposed TOF estimation method works well on the received signal with a low SNR and improves the ranging accuracy by about 5 mm when the blade tip distance is larger than 500 mm. This study provides a good reference for the accurate measurement of rotor blade tip distance, and gives a solution for ranging high-speed rotating objects.

## 1. Introduction

In the 1960s, the emergence of coaxial rotor helicopters not only broke through the limitation of the cruising speed of traditional single-rotor helicopters, but also had the advantages of small size, compact structure, high weight efficiency, enhanced stability, and good manoeuvrability [1,2,3,4], and were widely used in civilian applications such as firefighting, search and rescue, etc. However, the dual-rotor system in coaxial helicopters poses a risk of collision between the contra-rotating upper and lower blades during flight. To mitigate this risk, one of the solutions is to continuously monitor the real-time distance between the blade tips at the moment of rotor intersection. This monitoring not only prevents collisions, but also aids in optimising rotor spacing for improved performance.

David H. Hunter et al. [5] proposed a method to monitor the blade tip distance of a coaxial rotor using wireless measurement technology, which is achieved by embedding electric or magnetic field antennas inside the upper and lower rotor blades using the principle of near-field effect [5]. However, this method is still in the theoretical research stage and the experiments in principle or actual engineering experiment verification have not been reported. Stereo vision measurement is also widely used to measure blade deflection and motion [6,7,8]. However, this method requires mounting the cameras under the rotor and can be difficult to accurately measure the blade tip distance between the contra-rotating upper and lower rotor blades due to the blocking view of each other. Fibre optic sensing was also proposed to monitor blade deformation [9,10], where a series of fibre optic sensors were arranged on the surface of the blade, and the strain deflection line of the blade was obtained by fitting and decoupling the strain of the sensors. Due to the discrete distribution of fibre optic sensors, the installation angle has a great influence on the calculation result, and the decoupling of strain is difficult [10]. Research on electromagnetic ranging based on FMCW millimetre-wave radar is ongoing [11]; however, it can be difficult to receive adequate echo signal from small-sized rotor blades, e.g., chord length in tens centimetres, during dynamic measurement. Electromagnetic ranging also suffers from low resolution, e.g., 37.5 mm in resolution with the commonly used electromagnetic bandwidth of 5 GHz [12], as well as the risk of electromagnetic interference. 

Ultrasonic ranging methods are widely utilised in various industries for distance measurement applications. One of the most common ultrasonic ranging methods is the time-of-flight (TOF) technique [13]. The TOF technique involves transmitting a burst of ultrasonic waves towards a target, and the propagation time is recorded for the distance calculation. The TOF method is known for its accuracy and reliability in providing precise distance measurements [14]. Another popular ultrasonic ranging method is the phase shift (PS) technique [15], where the phase difference between the transmitted and received ultrasonic waves is measured to determine the distance to the object. The PS method is advantageous in noisy environments or when multiple reflective surfaces are present [15]. Recent advancements in ultrasonic ranging technology have led to the development of more sophisticated methods such as frequency-modulated continuous wave (FMCW) ranging [16]. FMCW ranging utilises a continuous wave of ultrasonic waves with varying frequencies to measure distance based on the frequency shift of the reflected waves, offering improved range resolution and accuracy compared to traditional pulse-echo methods.

Considering the requirements of an accurate short-distance measurement for blade tip distance in coaxial rotor helicopters (usually 100–1000 mm), ultrasonic ranging based on TOF emerges as a suitable option. Ultrasonic ranging sensors can be conveniently embedded in the rotor blades without significantly affecting the overall aerodynamics of the helicopter. Unlike other methods, ultrasonic ranging is non-contact, eliminating the risk of physical interference and damage to the blades. It is also unaffected by electromagnetic interference, ensuring reliable measurement in the presence of electrical equipment or electromagnetic fields. Furthermore, ultrasonic ranging provides a fast response time, enabling the real-time monitoring and immediate detection of potential collision risks between the contra-rotating blades. However, compared with the high-speed rotation of the coaxial rotor helicopter blades, i.e., 300–600 revolutions per minute (RPM), the propagation speed of ultrasonic waves in the air is relatively slow, which can lead to missing the effective echo signal by the receivers during the short-period intersection of the blades. Moreover, the environmental noise is complex during the actual flight of the helicopter, which leads to the low signal-to-noise ratio (SNR) of the received signal; therefore, accurate ranging results cannot be obtained by using the traditional TOF estimation method.

In this paper, we propose a coaxial rotor tip distance measurement method based on an ultrasonic measurement window and phase triggering. The ultrasonic transmitting and receiving transducers are mounted near the tip of the upper and lower rotor blades, respectively, and the ultrasonic emission transducers are triggered by the phase information of the known phase measurement system. To cope with low SNR received signal, a TOF estimation method based on signal power density is utilised. Then, the dynamic measurement of the blade tip distance is carried out on an experiment platform with single-element transducers.

This paper is structured as follows: Section 2 gives the methodology of blade tip distance measurement; Section 3 introduces the specific signal denoising method, TOF estimation method, and sound velocity compensation method; Section 4 presents the details of the experiments and the discussion on the results of signal denoising, dynamic ranging with single-element transducers; and Section 5 concludes the experiments and provides the future direction of the research.

## 2. Distance Measurement Method

### 2.1. Sensor Arrangement and Measurement System

Most ultrasonic ranging methods are based on the principle of reflection. However, the ranging accuracy can be compromised because of the distortion and/or overlapping of the echo signal caused by the various conditions of the reflective objects, e.g., angled surface, multiple-layer structure, close-by objects, etc. [15]. After considering the size of the reflective surface of the rotor blades and the high speed of the contra-rotating nature, the pitch-catch ultrasonic ranging mode based on the separated transmitter and receiver was utilised. This ranging mode can reduce the dead zone of the ranging and halve the attenuation of ultrasound in the air compared to transmitting and receiving with the same ultrasonic transducer [14].

**Figure 1:**
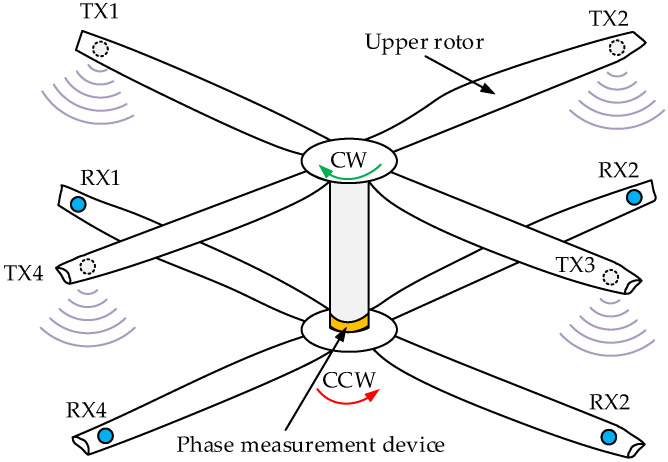
Locations of the transmitters (TX), receivers (RX), and a phase measurement device on coaxial rotors. CW—clockwise, CCW—counterclockwise.

As shown in Figure 1, the ultrasonic transmitters facing downward are individually installed near the tip of the upper rotor blades, and the receivers facing upward are installed near the tip of the lower rotor blades. Due to the short period of the intersection between the upper and lower rotors, e.g., a couple of milliseconds, a phase measurement device is installed on the coaxial axis to trigger the transmitters before the two blades intersect. This ensures that the transmitted ultrasound has enough time to propagate and meet the receiver.

**Figure 2:**
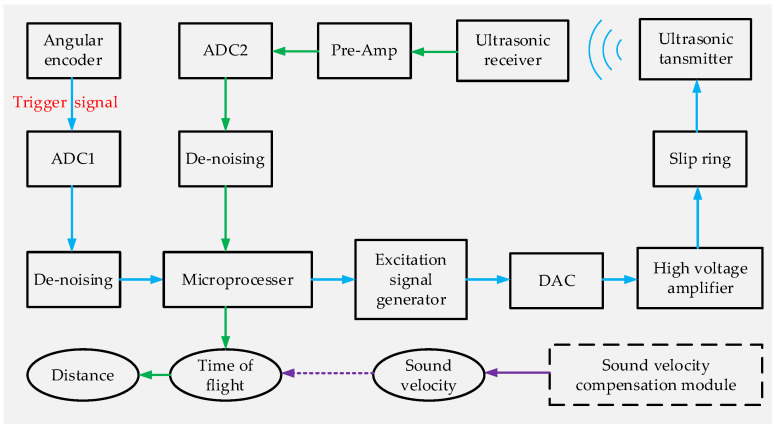
A block diagram of the proposed blade tip distance measurement method.

Figure 2 shows a block diagram of the proposed method. The measuring system is composed of a sensing module, a signal processing module and a sound velocity compensation module. Xilinx’s artix7 series FPGA is used as the microprocessor. After acquiring the trigger signal generated by the angular encoder at a preset trigger phase, the microprocessor generates an excitation signal via the DDS (direct digital frequency synthesis) method. Then, the sensing module amplifies the excitation signal and transmits it through the slip ring to the ultrasonic transmitter. After the ultrasonic wave is propagated in the air, the ultrasonic receiver receives the signal and amplifies it through the preamplifier. The signal processing module includes the AD/DA converters of the transmitted and received signal, the de-noising of the received and trigger signal, and the distance calculation. The sound velocity compensation module obtains the real-time sound velocity by ranging a fixed-distance target with a calibrated ultrasonic sensor. This compensation method can also compensate for the errors caused by various environmental factors, e.g., temperature and humidity. The final distance is calculated with the real-time sound velocity and TOF by the microprocessor.

### 2.2. The Conception of Ultrasonic Distance Measurement Window

**Figure 3:**
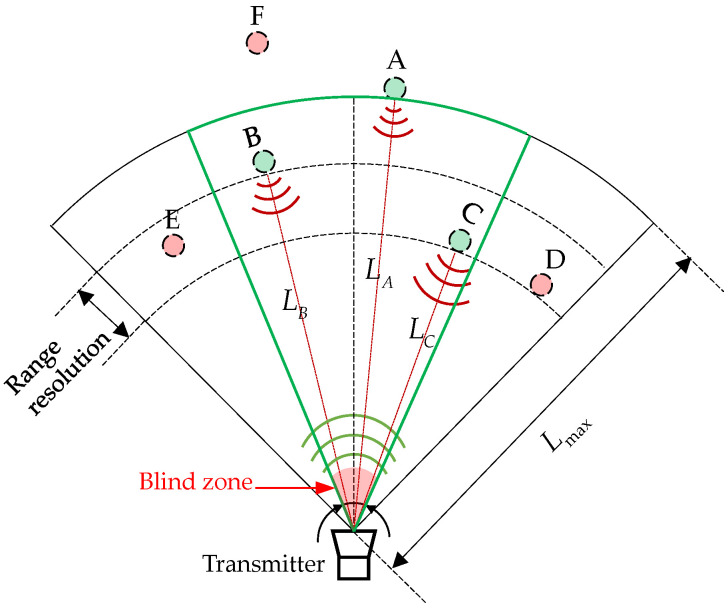
Schematic 2D representation of the effective ranging area. Receivers A, B, and C can obtain the distance measurement value, while receivers D, E, and F cannot obtain the ranging result because they are outside the effective ranging angle range.

The directivity of ultrasonic transducers is a physical property that describes the distribution of sound pressure intensity in different directions [17]. The directivity function determines the angular pressure distribution (usually normalised) for any radial location and can be measured experimentally or derived theoretically [18,19,20]. The magnitude of pressure diminishes usually inversely with the radial distance [20]. The directivity has a great effect on the sensitivity and accuracy of ultrasonic ranging [21]. Ultrasound beamwidth refers to the angle at which the directivity function of the main beam drops to 0.707 of the maximum value. Generally, the smaller the beamwidth, the sharper the directivity of the sensor. The directivity of ultrasonic ranging is also related to the resonant frequency and the radiation area of the sensor [22]. Due to the directivity of ultrasonic wave propagation and the dependence of ultrasonic energy on the beam angle range, the effective angle for ultrasonic ranging is limited. Figure 3 shows a schematic representation of the effective ranging angle resulting from the directivity of an ultrasonic transducer. The green sector with a central angle, θ, indicates that the trustworthy distance value can be obtained when the receiver is located in this region. A blind zone for the pitch–catch mode is mainly caused by the near-field zone of the transmitter. 

**Figure 4:**
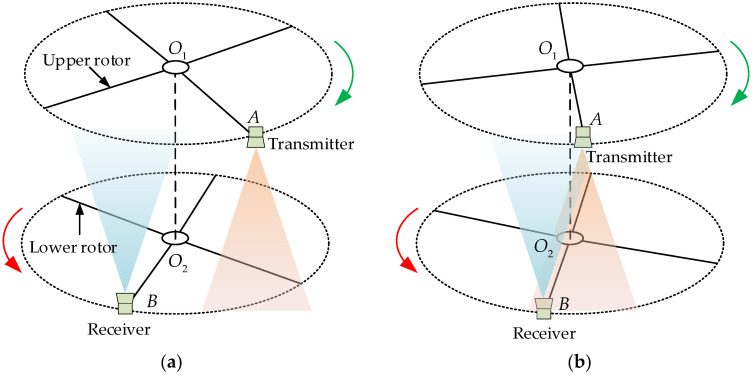
Positions of the transmitter and the receiver, (**a**) no valid ultrasound signal can be received outside the measurable window, and (**b**) a valid ultrasound signal can be received inside the measurable window. Where $O_{1}O_{2}$ is the coaxial line, and $O_{1}A$ and $O_{2}B$ are the upper and lower blade lines, respectively.

Because of the positive correlation between the effective ranging area and the beam angle [23], the concept of a measurement window is constructed by utilising the beam angle. The ultrasonic measurement window here refers to the spatial range within which the receiver can receive the ultrasonic signal within the effective ranging area of the transmitter during the intersection of the upper and lower blades, that is, to ensure that the receiver can achieve an effective measurement within the measurement window. The basic model of ultrasonic ranging based on the measurement window is shown in Figure 4. At the state when the effective ranging area of the transmitter and receiver are not overlapped (Figure 4a), no valid ultrasound signal can be received. When the upper and lower blades continue rotating to have the beam angles overlap (Figure 4b), the receiver can receive a valid ultrasound signal. After completing the signal reception at the duration of the intersection, the received ultrasound signal is processed to obtain the distance.

### 2.3. Trigger Timing

**Figure 5:**
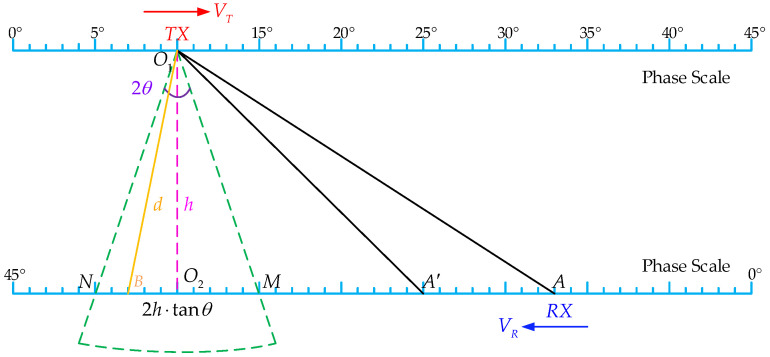
A schematic diagram of calculating the timing of triggering.

As mentioned in Section 2.1, to prevent missing the receiving signal when the blades meet, the transmitter must be triggered in advance based on the phase information from the angular encoder. Taking the eight-blade helicopter shown in Figure 1 as an example, the phase interval between the two adjacent blades’ intersection is 45°. Converting the rotating surface into a plane, as shown in Figure 5, the calculation method for triggering timing is as follows. 

If the measurable window angle of the transmitter is 2θ and the vertical spacing between the transmitter and receiver is h, then $\left|MN\right|=2h\cdot \tan\theta$, and the velocities of the transmitter and the receiver are $V_{T}$ and $V_{R}$, respectively. When the transmitter moves to point $O_{1}$ and the receiver moves to point A, the transmitter is triggered to emit ultrasonic waves. 

On the one hand, to ensure that the receiver can reach point M before the wave passes through MN, the following equation must be satisfied:

(1) $$T_{A\to M}\leq ToF$$

where $T_{A\to M}$ is the time duration of the receiver from point *A* to *M*, and ToF is the propagation time of the ultrasound. Let the speed of sound be c, then:

(2) $$\frac{\left|AM\right|}{V_{R}}\leq \frac{h}{c\cdot \cos\theta }$$

On the other hand, to ensure that the wave can reach MN before the receiver moves past point N, by the same token, the following equation must be satisfied:

(3) $$\frac{h}{c\cdot \cos\theta }\leq \frac{\left|AM\right|+2h\cdot \tan\theta }{V_{R}}$$

which corresponds to the trigger moment, i.e., when the receiver is at point $A^{\prime }$.

To sum up, the earliest trigger time should not be earlier than point *A*, and the latest trigger time should not be later than point $A^{\prime }$, otherwise, the receiver cannot receive the signal. Combining Equations (2) and (3):

(4) $$\frac{h\cdot V_{R}}{c\cdot \cos\theta }-2h\cdot \tan\theta \leq \left|AM\right|\leq \frac{h\cdot V_{R}}{c\cdot \cos\theta }$$

**Figure 6:**
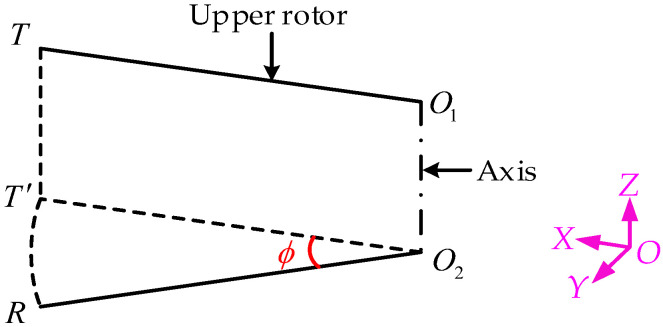
A schematic diagram of calculating the phase difference.

Then, we can determine the trigger timing by the phase difference ϕ between the transmitter and receiver through $\left|AM\right|$, as shown in Figure 6, where $T^{\prime }$ is the projection of *T* on the rotating plane of the lower rotor.

(5) $$\phi =\frac{\overset{⏜}{T^{\prime }R}}{O_{2}R}=\frac{\left|AM\right|+h\cdot \tan\theta }{O_{2}R}$$

To prevent the receiver from missing the signal, analytic Equations (4) and (5) show that θ must be large enough, and the trigger timing of the transmitter is a range value under a certain rotational speed, with the boundaries of the range determined by the range of the distance to be measured. The following is an example of determining the trigger timing.

For demonstration, the parameters of a helicopter rotor are assumed as follows: the rotor radius is 4.0 m, the rotation speed is 400 RPM, and the receiver is mounted at a distance of 210 mm from the blade tip; then, the linear speed of the receiver is about 158.75 m/s. When the sound velocity (331.4 m/s) is taken as zero degrees Celsius, for the measurement range of the blade tips, *h =* 100~1000 mm, it can be obtained from Equations (4) and (5) that θ must be greater than 23.2° and $0^{{}^{\circ}}<\phi \leq 1.44^{{}^{\circ}}$ is required under $\theta =23.2^{{}^{\circ}}$. It is also worth noting that the measured distance, *d*, as shown in Figure 5, is an approximation of the vertical distance between the blade tips.

To verify the proposed method, an experimental test platform is set up with the parameters as follows: $O_{2}R$ is 0.9 m, the rotation speed of the transmitter is 480 RPM, and the receiver is mounted away from the transmitter at various distances, 100–1000 mm. The measurement window angle of the transducer used during the test is about 14°, and the sound velocity is about 340 m/s. Plugging in these parameters, $0.81^{{}^{\circ}}\leq \phi \leq 1.84^{{}^{\circ}}$ is required to perform a successful measurement.

## 3. Signal Processing

### 3.1. Denoising

The ultrasonic received signal is contaminated with electronic noise and irrelevant clutter signals. When the detection distance is long, the SNR of the received signal is extremely low, which seriously affects the ranging accuracy. Therefore, appropriate denoising methods are needed to improve the ranging accuracy.

Bandpass filters are commonly used for denoising ultrasonic received signals. To design a suitable bandpass filter, some evaluation indicators, such as the signal-to-noise ratio (SNR), mean square error (MSE), and normalised cross-correlation (NCC), can be used to evaluate the filtering effect of different filter parameters. However, to obtain these indexes, it is necessary to first establish a standard model for the received signal. 

**Figure 7:**
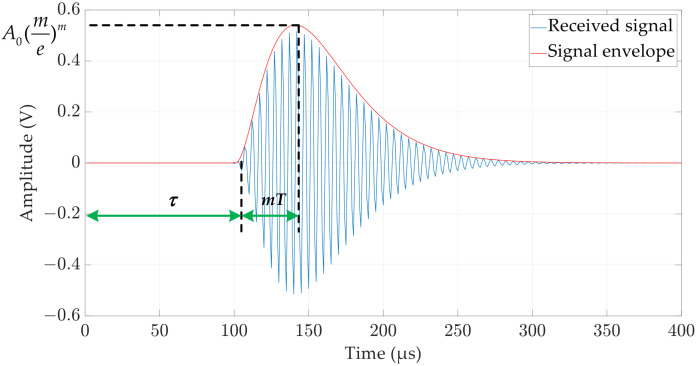
A representation of a standard ultrasonic signal at reception, generated using the mixed exponential decay model.

Narrow-band ultrasound is often used in ultrasonic ranging systems, and its received signal can be described by a mixed exponential model [24,25]:

(6) $$s(t)=A_{0}{\left(\frac{t-\tau }{T}\right)}^{m}e^{-\frac{t-\tau }{T}}\sin[2\pi f_{c}(t-\tau )+\varphi ]$$

where $A_{0}$ is the amplitude factor, T and m correspond to the transducer performance (1<m<3), τ is the starting point of the waveform, $f_{c}$ is the transducer centre frequency, and φ represents the phase (generally 0 is used). Figure 7 shows the representation of the received signal generated by Equation (6), based on the mixed exponential empirical model. It can be seen that the peak $A_{0}{\left(\frac{m}{e}\right)}^{m}$ occurs when t=τ+mT. m is selected to be 2 to obtain a close match between the representation and the actual received signal in this study. 

**Figure 8:**
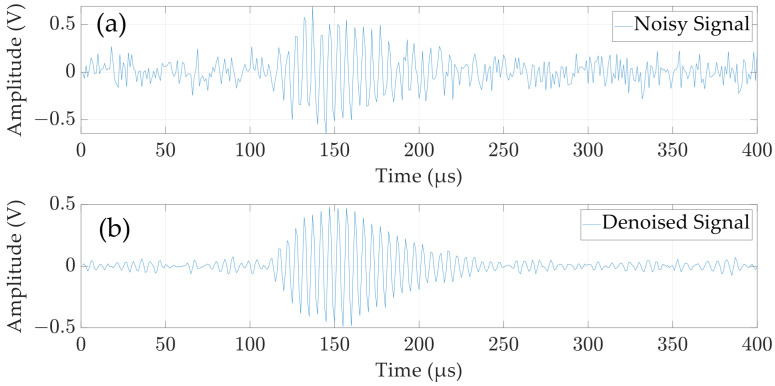
Simulation graph of the filtering effect of the designed bandpass filter. (**a**) Noisy signal; (**b**) Denoised signal.

The Butterworth filter is well-suited for designing bandpass filters due to its flat passband response and steep stopband attenuation [26]. The passband bandwidth of the bandpass filter is determined by the effective bandwidth of the received signal. Considering the real-time requirements of the blade tip distance measurement, it is advisable to keep the filter order low. Based on these considerations, this study establishes a mixture exponential decay model (Equation (6)) for the received signal and adds Gaussian white noise to evaluate the filtering effect of different filter parameters. Ultimately, a first-order Butterworth bandpass filter is designed with passband starting and ending frequencies of 181 kHz and 219 kHz, respectively. And the evaluation results of the filtering effect of the designed filter are shown in Table 1 and Figure 8. Usually, the larger the SNR and NCC, and the smaller the MSE, the better the denoising effect [27].

### 3.2. Distance Calculation

#### 3.2.1. TOF Estimation

**Figure 9:**
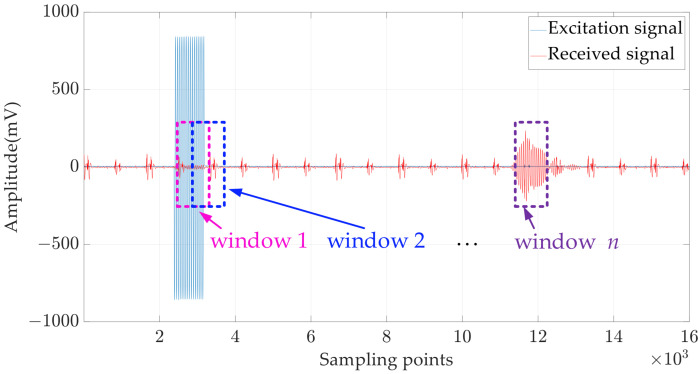
Signal and window exemplars for the sliding window power density method.

The common TOF estimation methods include the amplitude threshold method (ATM), envelope fitting method and cross-correlation method [28,29,30], where the correlation approach is considered statistically optimal [31,32] because it uses the entire phase and amplitude information contained in the signal [33]. However, these methods all have SNR requirements for the received signal and are therefore not suitable for the current application in which the received signal has a poor SNR in general. Hence, a TOF estimation method, the sliding window power density method (SWPDM), is utilised to process the received ultrasound signals with a low SNR. As shown in Figure 9, the proposed method should be carried out according to the following steps:

(1) Select the window width *N* and sliding unit step *step*. The window width depends on the typical length of the received signal, and the sliding unit step should not be set too large (set as 1 in this study).
(2) Determine the start and end point of the window. The start point depends on the trigger signal of the angular encoder and the end point is decided by the farthest measurement distance.
(3) Calculate the power density of the received signal in windows 1 to n.
(4) Take the serial number of the window with the highest power density, written as n.
(5) Determine whether the window with the highest power density is significantly larger than the window where the general noise is located and record the window serial number if it is larger; otherwise, it is considered that there is no received signal in this measurement. In this study, the definition of whether it is significantly greater is whether the power density of the window in which the maximum power density is located is greater than 2 times the average of all windows.
(6) Calculate the TOF using the following equation:

(7) $$ToF=\frac{n\cdot step}{f_{s}}$$

where $f_{s}$ represents the sampling rate of the received signal.

Analysis of the algorithm flow above reveals that the theoretical ranging error of the SWPDM primarily depends on the window width, sliding unit step, and threshold selection. Both excessively large and small window widths can lead to increased ranging errors, so it is recommended to match the window width with the typical length of the received signal in the practical application. As the sliding unit step increases, the ranging error also increases, but the measurement cycle for each measurement becomes smaller, thus requiring a compromise selection. Additionally, inappropriate threshold selection can result in distance measurement errors, so it is necessary to choose an appropriate threshold method based on the specific application. 

**Figure 10:**
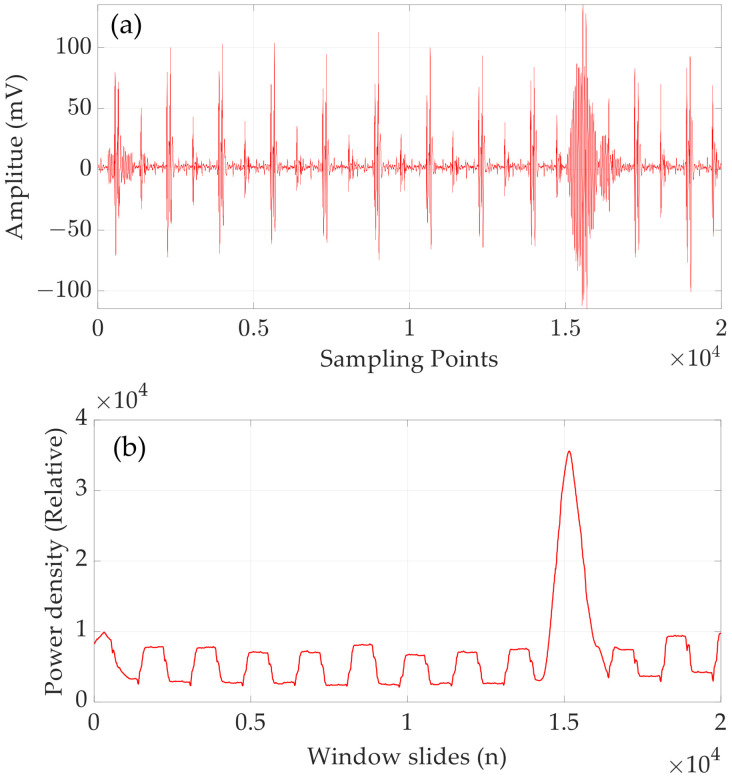
(**a**) A typical received signal with a low SNR. (**b**) Power density of all sliding windows.

A typical low SNR received signal is shown in Figure 10a, which was acquired with a data acquisition card in a dynamic experiment. It is not difficult to see that it is difficult to estimate the start time of the received signal if the amplitude threshold method or the cross-correlation method is used, but when the SWPDM is used, it can be estimated very accurately. Figure 10b shows the power density plot for all sliding windows. The window where the maximum power density is located is the window where the received signal is located, so we can estimate the TOF by counting the number of sliding windows.

#### 3.2.2. Ultrasonic Velocity Compensation

**Figure 11:**
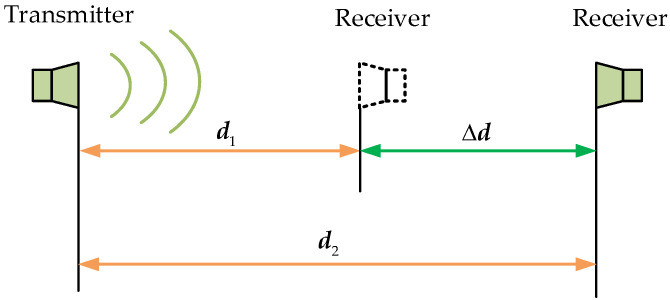
A schematic diagram of the sound velocity compensation method.

The speed of sound in the air is affected by many factors such as air temperature, humidity, and pressure [34,35]. Here, an indirect sound speed compensation method is used to obtain real-time sound velocity in a complex environment. As shown in Figure 11, the receiving transducer is first placed at the distance from the transmitting transducer $d_{1}$, and the measured TOF is $∆T_{1}$. Then, the receiving transducer is moved to a distance $d_{2}$, and the TOF at this time is measured to be $∆T_{2}$. Then, the compensated sound velocity c can be represented by Equation (8):

(8) $$c=\frac{d_{2}-d_{1}}{∆T_{2}-∆T_{1}}=\frac{∆d}{∆T}$$

where ∆d is acquired by a high-precision mobile platform, as shown in Figure 10. In general, the accuracy of the measured sound velocity depends on the accuracy of the high-precision moving platform and the TOF estimation. Here, by calculating the velocity from the changes in distance and time, the overall effect of the errors from the moving platform and TOF estimation is minimised. In this study, a motorised lead screw guide has an accuracy of 0.03 mm. 

## 4. Experiments and Result Discussion

### 4.1. Experiment Set-Up

**Figure 12:**
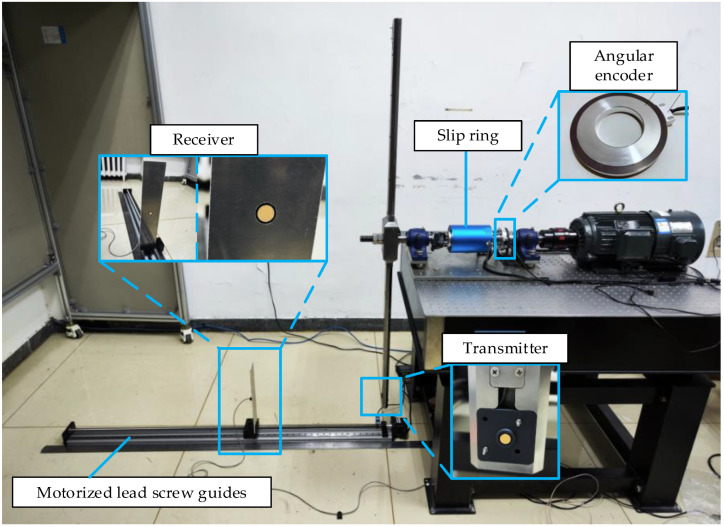
The set-up of dynamic ranging experiments.

The dynamic ranging test platform of the blade tip distance is shown in Figure 12. The ultrasonic transmitter is embedded in the position of the blade tip of the simulated rotor. The receiver is fixed on the electric lead screw guide rail, which can be moved on the guide rail. The stroke of the lead screw guide is 1.2 m, and the distance accuracy is 0.03 mm. Considering the centre frequency, directionality, ultrasonic propagation characteristics, and size of the ultrasonic transducer, both the transmitter and receiver are the DYA-200-01B ultrasonic transducer (Hangzhou Umbrella Automation Technology Co., Ltd., Hangzhou, China) with a centre frequency of 200 kHz and a beam angle of 14.5°. And it was chosen mainly for four reasons: (a) the high centre frequency, for ultrasonic ranging, theoretically gives a better resolution and accuracy, as long as it suits the detection range in the air; (b) the size, which is small enough to be embedded in the rotor blades of coaxial helicopters without affecting the aerodynamic performance of the rotor blades; (c) the transducer’s beam angle of 14.5° is large enough to meet the 2θ requirement calculated in Section 2.3; (d) the market availability. The excitation signal of the transmitter is a 200 kHz, 200 Vpp sine wave, and each single excitation lasts for 16 cycles. In addition, 200 Vpp is used to ensure that the amplitude of the received signal energy from a distance of 1000 mm is sufficient under different weather conditions for the proposed application. When the AD module of the microprocessor detects the trigger signal of the angular encoder, the DA module sends out an excitation signal, which is amplified by the high-voltage amplifier and acts on the transmitter through a slip ring.

Before starting the dynamic tests, two fixed-distance measurements are performed at the distances of 300 mm and 500 mm. Then, the speed of sound compensation is performed, giving the compensated speed of 339.50 m/s. During the experiment, the aperture of the transmitter and the receiver are carefully aligned at distance zero first. Then, using the movement of the electric lead screw guide as the distance reference, each movement is 100 mm. The vertical distance (100–1000 mm) between the transmitter and receiver was measured with the blade rotated, and the blade speed was set to 480 RPM. The trigger timing was set to 1.5° according to the calculation in Section 2.3.

### 4.2. Results of the Ranging

#### 4.2.1. Signal Timing and Denoising

**Figure 13:**
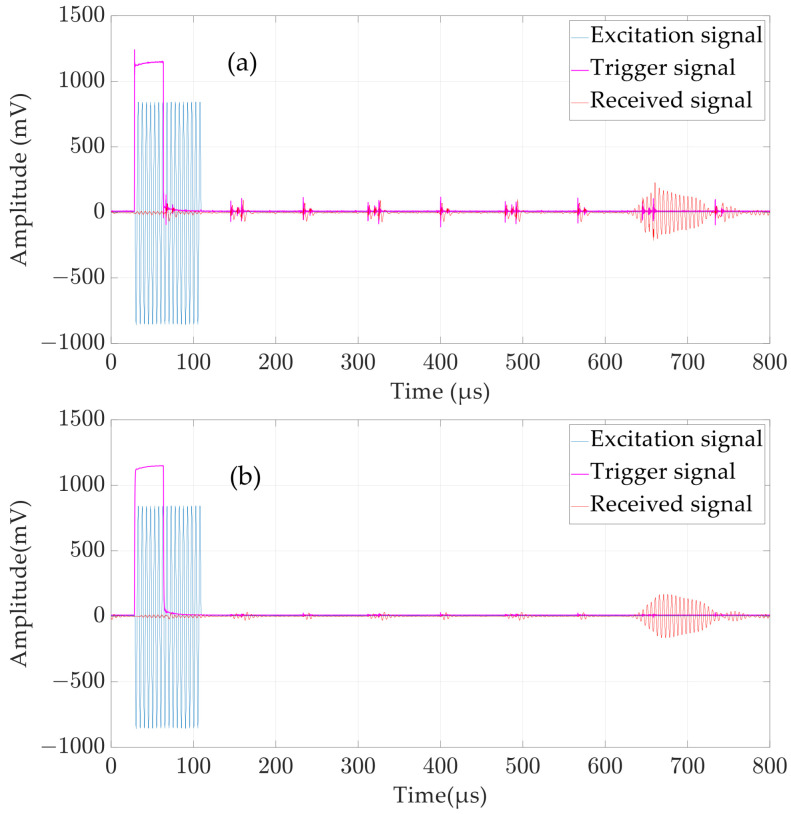
Typical signals for a single dynamic measurement: (**a**) signal before denoising; and (**b**) signal after denoising.

To verify the correctness of the signal timing and the effectiveness of signal denoising, in the dynamic ranging experiment, the typical signal raw data from a distance measurement are collected by a data acquisition card (Multi-channel synchronous acquisition card with a maximum sampling rate of 10 M/s, Chengdu MySoow Electric Co., Ltd., Chengdu, China), including the trigger signal from the angle encoder, the excitation signal of the transmitting transducer emitted by the DA module of the FPGA, and the reception of the receiving transducer, and then plotted using MATLAB, as shown in Figure 13. It should be noted that the received signal in Figure 13 is denoised using a bandpass filter designed in Section 3.1, while the triggering signal is filtered using a first-order Butterworth low-pass filter with a cutoff frequency of 500 kHz.

Figure 13 confirms that the signal timing is correct. Comparing Figure 13a,b, it can be seen that our denoising method is effective, and the signal-to-noise ratio (SNR) of the trigger signal and the received signal is improved, which is conducive to improving the stability and accuracy of the ranging system.

#### 4.2.2. Ranging Results and Error Analysis

To evaluate the TOF estimation method proposed in this article, comparative experiments are conducted using different TOF estimation methods mentioned in Section 3.2.1. The specific operation of the amplitude threshold method (ATM) is to first locate the peak of the received signal and then, using the peak position as a reference, move backwards by 45 μs (half of the typical length of the received signal) to determine the starting time of the received signal. And the cross-correlation method (CCM) selects the transmit signal as the reference signal. In cases where the distance is far, the received signal exhibits a low SNR and multiple peaks even after filtering, making it difficult for the envelope fitting method (EFM) to accurately complete the envelope fitting. Therefore, it is not suitable for the application environment in this study. 

**Figure 14:**
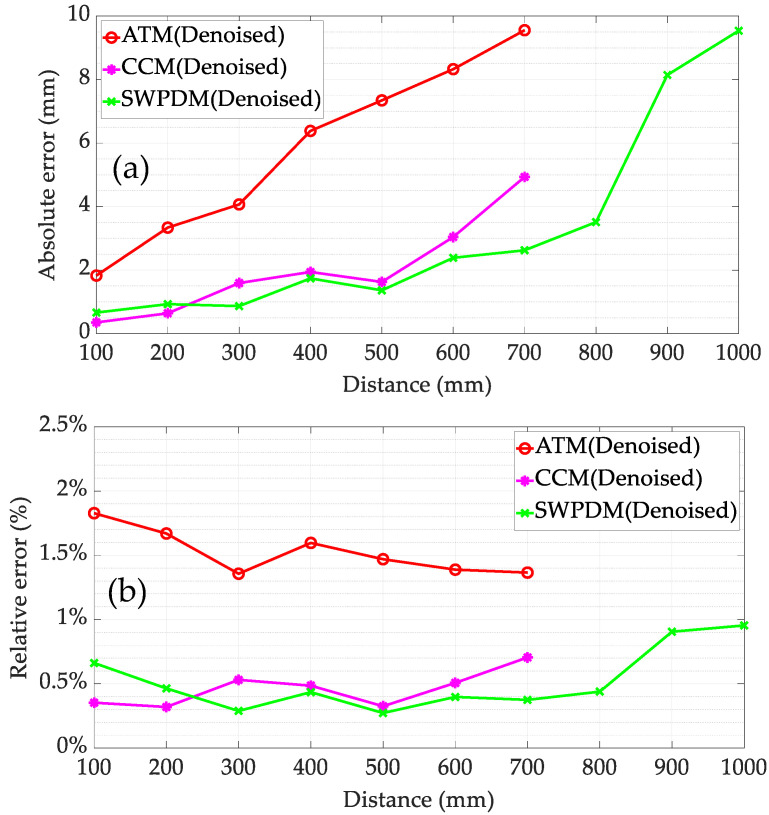
Maximum ranging error of different TOF methods: (**a**) the absolute errors; (**b**) the relative errors.

Figure 14 displays the maximum dynamic ranging errors of ATM, CCM, and the proposed TOF estimation method (SWPDM) within the distance range of 100–1000 mm. Beyond 700 mm, although ATM and CCM can still provide distance measurements, the results become highly unstable and exhibit significant errors, rendering them unsuitable for helicopter rotor collision warning systems; hence, they are not included in Figure 14. Generally, the absolute error for all three methods increases with distance due to the attenuation of ultrasound in air resulting in a lower SNR of the received signal. Both SWPDM and CCM outperform ATM in terms of accuracy. In the case of SWPDM with denoised signals, the maximum error (9.54 mm, 0.96%) occurs at 1000 mm, and within the measurable distance range, SWPDM’s accuracy is generally on par with CCM. Overall, SWPDM is better equipped to handle low SNR received signals and is more likely to cope with the more complex dynamic noise environment of actual coaxial helicopters. Moreover, CCM’s reliance on cross-correlation operations results in a higher time complexity compared to SWPDM based on addition and absolute value operations, making SWPDM more suitable for helicopter environments requiring real-time measurements. 

**Figure 15:**
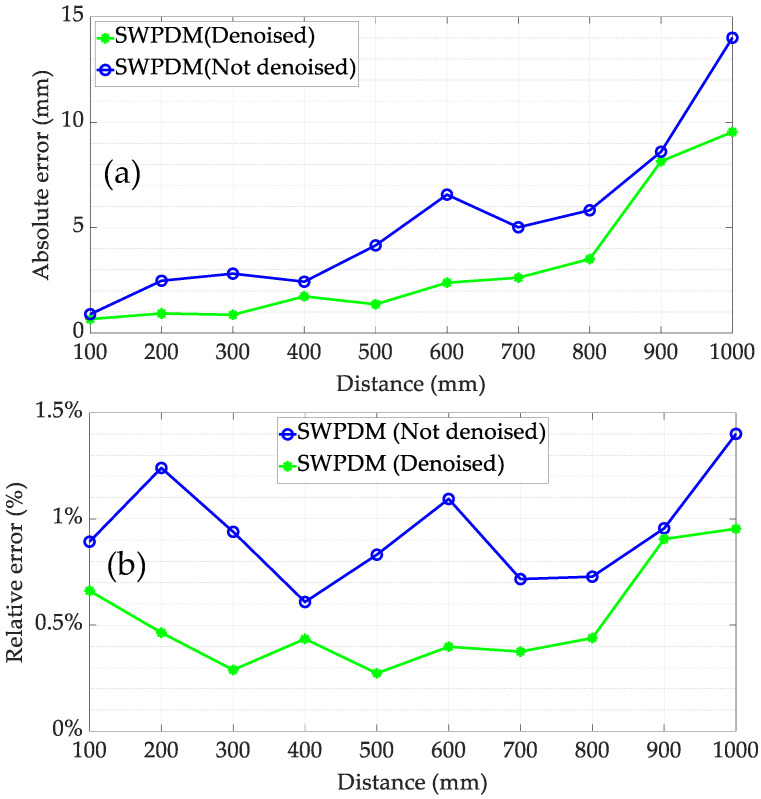
Maximum ranging error of SWPDM: (**a**) the absolute errors; (**b**) the relative errors.

To further analyse the impact of SNR on the proposed method in this article, comparative experiments were conducted between receiving signals with and without noise reduction. The experimental results are presented in Figure 15. The analysis demonstrates that the proposed method can achieve superior measurement results compared to ATM even under the conditions of a low signal-to-noise ratio (SNR). Furthermore, when the SNR is enhanced through filtering, the proposed method can enhance the ranging accuracy to a certain extent. For example, the maximum relative error decreased from 1.4% to 0.96%. 

Furthermore, some typical normal evaluation indices such as the root mean square error (RMSE), absolute mean error (MAE), and standard deviation are calculated and presented in Table 2. The analysis reveals that while the proposed method offers the advantage of low requirements for the SNR, enhancing the SNR is also beneficial for improving the ranging accuracy and stability. Compared to ATM, both SWPDM and CCM exhibit superior accuracy and stability. Although CCM and SWPDM perform similarly, as previously mentioned, CCM faces challenges in achieving stable measurements over long distances and has a higher time complexity. Therefore, SWPDM emerges as the preferable choice.

The analysis of the results shows that the accuracy of the method presented in this study can be lower than that of traditional ultrasonic distance measurement applications. However, the key advantage of this method lies in its suitability for application in high-speed dynamic environments compared to other blade tip distance measurement methods, such as the monocular vision-based approach [36], which gives an error margin with a maximum error of 1.99 mm. Nonetheless, the visual method faces challenges, including potential obstruction of the camera’s field of view by the helicopter body and its limited effectiveness in varying weather conditions, restricting its application to experimental stages. The method proposed in this study not only meets the accuracy requirements for blade tip distance measurement, but also shows promise for practical application in real coaxial rotor helicopters.

## 5. Conclusions and Future Work

This paper presents a method based on an ultrasonic measurement window and phase triggering for the distance measurement of the contra-rotating rotor blades on coaxial rotor helicopters. The feasibility of this method is studied through the experiments performed on a laboratory simulation test platform. The experimental results show that the angular encoder installed on the rotating axis can trigger the ultrasonic emission module well, and the proposed TOF estimation method has the advantage of extracting the ultrasound TOF from the received signal with a very low SNR, which is hard to realise by other TOF estimation methods. In future work, the stability of receiving effective signal during the blades’ intersection can be improved by expanding the ranging window of the ultrasonic sensors, e.g., by using ultrasonic array transducers; however, this may also bring in challenges caused by acoustic field interference between different array elements.

## Figures and Tables

**Table 1:** Evaluation of denoising effect.

Index	SNR	MSE	NCC
Before denoising	2.1602	0.011374	0.77842
After denoising	9.0296	0.002338	0.93607

**Table 2:** Evaluation of the normal indexes of ranging errors (100–700 mm).

TOF Method	RMSE	MAE	S *
ATM (denoised)	4.6279	3.6611	3.4562
CCM (denoised)	2.9036	2.3249	1.3076
SWPDM (denoised)	2.6736	2.2586	1.1585
SWPDM (not denoised)	3.4432	2.3493	2.4004

* The standard deviation.

## Data Availability

Data are contained within the article.
